# Low-dose palliative radiotherapy for malignant peripheral T-cell lymphoma masked by cellulitis and osteomyelitis: a case report

**DOI:** 10.1093/bjrcr/uaad010

**Published:** 2023-12-18

**Authors:** Yasir Alayed

**Affiliations:** Radiation Oncology Unit, Department of Medicine, College of Medicine, King Saud University, Riyadh 12372, Saudi Arabia

**Keywords:** recurrent lymphoma, low-dose radiotherapy, palliative, infection

## Abstract

Classic Hodgkin lymphoma is a potentially curable disease. With the advent of effective systemic regimens with adriamycin, bleomycin, vincristine, and dacarbazine, chemotherapy has become the treatment of choice for advanced Hodgkin lymphoma. However, for advanced Hodgkin lymphoma after chemotherapy, disease relapse rates are still high. This case report highlights how low-dose palliative radiotherapy can be used successfully for the management of an unusual case of recurrent lymphoma with a different histology soon after completing systemic therapy, which was further complicated by an ongoing local infection.

## Case presentation

A 33-year-old female with a 12-week history of progressive lower abdominal and back pain was referred to our outpatient haematological oncology department. She was previously healthy (ECOG PS 1) but had a 6-month history of weight loss, night-time sweats, and fever. A large inguinal mass measuring 3 cm was observed with tenderness and swelling upon physical examination.

## Investigations

Standard blood tests after admission revealed that white blood cells count was 29.0 × 10^9^ cells/L (reference values: 4.0 × 10^9^-11.0 × 10^9^ cells/L), with a neutrophil rate of 86.7% (reference values: 40%-75%), lymphocyte rate of 5.8% (reference values: 20%-45%), and monocyte rate of 4.8% (reference values: 3%-9%); haemoglobin levels were 108 g/L (reference values: 120-160 g/L); and platelet count was 541 × 10^9^ cells/L (reference values: 140 × 10^9^-450 × 10^9^ cells/L). Standard biochemistry studies revealed elevations in alkaline phosphatase (223 U/L, reference values: 40-150 U/L), gamma-glutamyl transferase (162 U/L, reference values: 5-55 U/L), total protein (90 U/L, reference values: 64-82 U/L), and blood urea nitrogen levels (6.9 mg/dL, reference values: 2.5-6.4 mg/dL). The patient had mildly lower albumin levels and an erythrocyte sedimentation rate of 120 mm (reference values: 0-24 mm). Hepatitis B surface antigen and the HIV were not detected in the viral screening.

Chest CT exhibited several enlarged lymph nodes in the supraclavicular, cervical, upper mediastinal, subcarinal, and right axillary regions. Abdominal CT revealed enlarged lymphadenopathy in the portocaval region as well as inguinal lymph nodes bilaterally, the largest seen on the left side measuring 2.5 cm. There was no splenomegaly on CT. A fluorodeoxyglucose-PET (FDG-PET)/CT scan showed avid lymphadenopathy in the above-mentioned areas, with the highest maximum standard unit value (SUVmax) of 15.3 in the left inguinal area.

A biopsy of the left posterior cervical and left inguinal lymph nodes revealed a nodular sclerosis subtype of classical Hodgkin lymphoma. The patient was given six cycles of ABVD/AVD chemotherapy, consisting of adriamycin, bleomycin, vincristine, and dacarbazine. The post-treatment PET scan (10 weeks post chemotherapy) showed complete metabolic resolution of the nodal disease above and below the diaphragm, with a Deauville score of 2.

**Figure 1.**
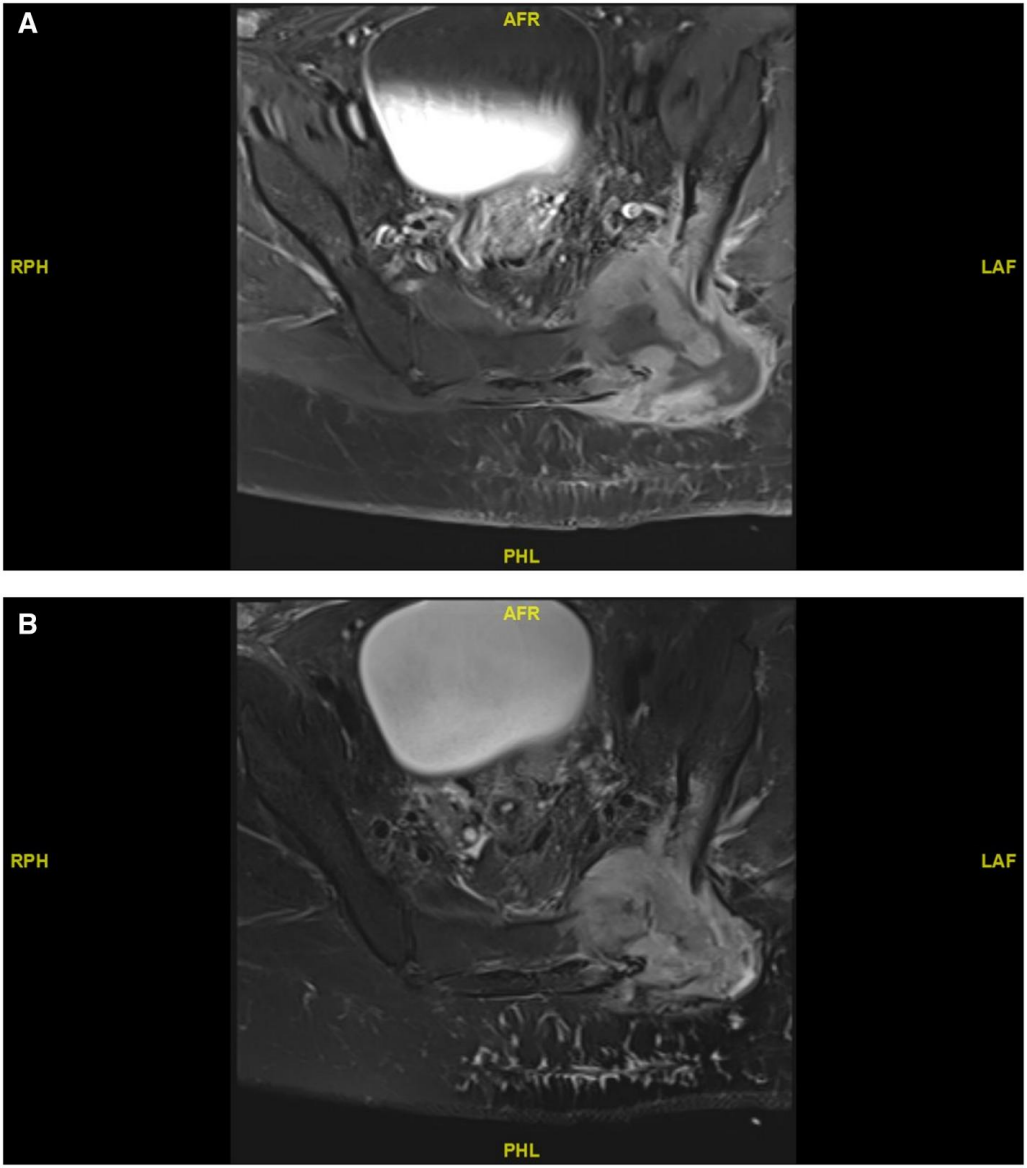
MRI axial (A) T1-weighted and (B) T2-weighted image showing osteomyelitis and cellulitis in the left gluteal region of the lower back.

Four months after chemotherapy, the patient complained of left gluteal pain with tenderness and swelling. She was febrile at the time. MRI revealed a subcutaneous collection extending to the left sacroiliac joint approximately 6 × 5 cm. There was extensive septic arthritis in the sacroiliac joint with adjacent bone osteomyelitis as well as diffuse subcutaneous oedema with cobblestone appearance in the left gluteal region suggesting cellulitis (Figure 1A and B). A PET scan was done at the time, which did not reveal any recurrence of her lymphoma. The clinical impression was an infection with osteomyelitis and cellulitis. Aspiration of the collection showed many pus cells, but cultures were negative.

## Treatment

The case was discussed in a multidisciplinary meeting, and the consensus was to proceed with wide-spectrum antibiotic coverage. The patient received piperacillin/tazobactam for 3 weeks, with minimal improvement in symptoms and MRI appearance. The case was again discussed in a multidisciplinary meeting, and the possibility of malignant involvement was raised. A left gluteal CT-guided biopsy revealed CD30-positive peripheral T-cell lymphoma, confirmed by a T-cell receptor polymerase chain reaction, which was a different diagnosis than her initial classical Hodgkin lymphoma.

**Figure 2.**
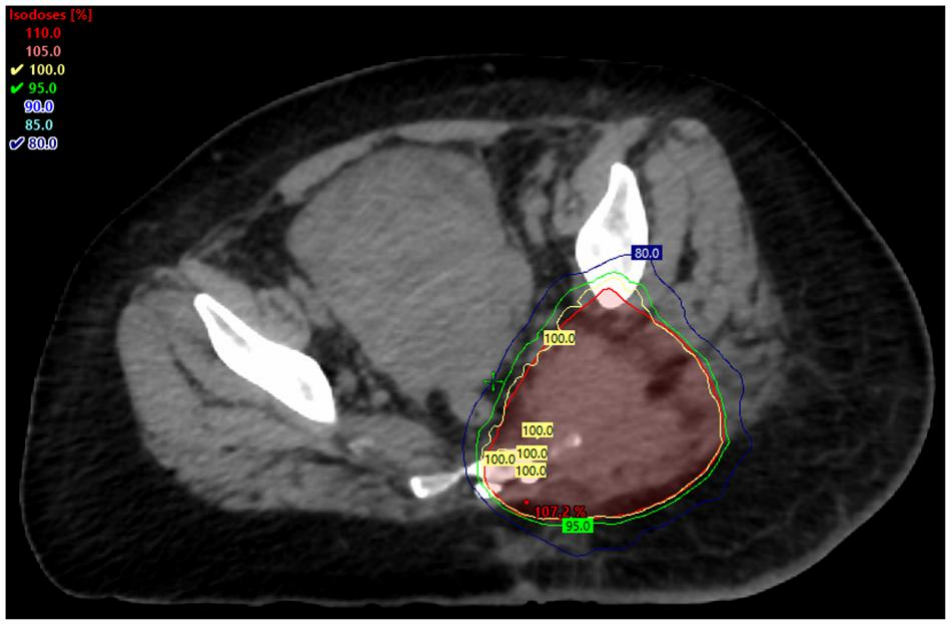
Axial view of the left gluteal region after palliative external beam radiation involving 4 Gy administered over two fractions using the volumetric modulated arc therapy technique.

At this stage, the patient was not clinically stable enough to undergo systemic therapy for her newly diagnosed T-cell lymphoma. Her gluteal pain was severe, and not adequately controlled medically. After discussing the case in a multidisciplinary meeting, palliative radiotherapy was suggested to relieve her pain until she was stable enough to proceed with definitive systemic therapy. She had volumetric modulated arc therapy, a form of external beam radiation, in which 4 Gy was administered in two fractions (Figure 2).

## Outcome and follow-up

The patient’s symptoms improved significantly within 1-week post-radiotherapy, and it was able to commence definitive systemic therapy as her general condition improved.

## Discussion

Although radiotherapy was the first effective treatment for Hodgkin lymphoma, advanced-stage (Stage III/IV) Hodgkin lymphoma is less curable with radiotherapy alone.^1^^,^^2^ Chemotherapy is now the preferred treatment for advanced Hodgkin lymphoma with the development of efficient systemic regimens such as ABVD.^2^^,^^3^ However, despite chemotherapy, disease relapse rates are still significant in advanced Hodgkin lymphoma.^4^ Studies have suggested that there might be a benefit for consolidation radiotherapy in select patients with advanced Hodgkin lymphoma.^4^^,^^5^ Occurrence of composite lymphomas is also not uncommon with classical Hodgkin lymphoma, and there are diagnostic challenges associated with composite lymphomas.^6^

This was a rare case of recurrence of a classical Hodgkin lymphoma that recurred as peripheral T-cell lymphoma within weeks after a complete response to chemotherapy. The fact that the patient had a recurrence so early was unusual and led to a belief that the painful gluteal collection was only an infection. Low-dose radiotherapy is a highly effective and safe treatment for the palliation of indolent lymphomas, but the role in more aggressive lymphomas is not as well established.^7^^,^^8^ The viability and excellent response to low-dose palliative radiotherapy in this patient were noteworthy, even in the context of ongoing infection in the area. In conclusion, low-dose palliative radiotherapy should be considered in symptomatic lymphoma patients as a bridge to systemic therapy in certain situations.

## Learning points

- The case presented illustrates an unusual case of recurrent lymphoma with different histology soon after undergoing systemic therapy, masked by cellulitis and osteomyelitis.
- With classical Hodgkin lymphoma, composite lymphoma occurrence is also not unusual and presents diagnostic challenges.
- Indolent lymphomas can be effectively and safely treated with low-dose radiotherapy, but their effectiveness in treating more aggressive lymphomas is less clear.
- Despite a persistent infection in the gluteal region, the patient had a favourable response to low-dose palliative radiation.
